# Happy creativity: Listening to happy music facilitates divergent thinking

**DOI:** 10.1371/journal.pone.0182210

**Published:** 2017-09-06

**Authors:** Simone M. Ritter, Sam Ferguson

**Affiliations:** 1 Behavioural Science Institute, Radboud University Nijmegen, Nijmegen, The Netherlands; 2 Creativity and Cognition Studios, Faculty of Engineering and IT, University of Technology Sydney, Sydney, Australia; Goethe-Universitat Frankfurt am Main, GERMANY

## Abstract

Creativity can be considered one of the key competencies for the twenty-first century. It provides us with the capacity to deal with the opportunities and challenges that are part of our complex and fast-changing world. The question as to what facilitates creative cognition—the ability to come up with creative ideas, problem solutions and products—is as old as the human sciences, and various means to enhance creative cognition have been studied. Despite earlier scientific studies demonstrating a beneficial effect of music on cognition, the effect of music listening on creative cognition has remained largely unexplored. The current study experimentally tests whether listening to specific types of music (four classical music excerpts systematically varying on valance and arousal), as compared to a silence control condition, facilitates divergent and convergent creativity. Creativity was higher for participants who listened to ‘happy music’ (i.e., classical music high on arousal and positive mood) while performing the divergent creativity task, than for participants who performed the task in silence. No effect of music was found for convergent creativity. In addition to the scientific contribution, the current findings may have important practical implications. Music listening can be easily integrated into daily life and may provide an innovative means to facilitate creative cognition in an efficient way in various scientific, educational and organizational settings when creative thinking is needed.

## Introduction

### Overview

Creativity is the driving force behind scientific, technological and cultural innovation, and it can be considered one of the key competences for the 21st century [1–2]. The problems we face in our complex and fast-changing world more than ever demand creative thinking. However, we are in a creativity crisis, people in general are thinking less creatively than before [3–5]. The question as to what facilitates creative cognition—the ability to come up with creative ideas, problem solutions and products—is as old as the human sciences [6]. Whereas in the Middle Ages creativity was reserved for those with divine inspiration, in the renaissance some humans, the genius minds, were finally thought capable of creative thought. Modern research has shown that creative thinking is inherent to normative cognitive functioning, rather than an innate talent available to only a few genius minds, and depends on fundamental cognitive processes [7–9]. Importantly, it has been demonstrated that creative cognition can be facilitated [10–11], and in the past decades various means to enhance creativity have been developed and tested (e.g., brainstorming, making random connections; for reviews, see [12–13]). The effect of music listening on creative cognition, however, has remained largely unexplored—despite earlier scientific studies demonstrating a beneficial effect of music on cognition [14] and an increased scientific interest in the influence of musical experience on cognitive functions unrelated to music abilities [15]. The current project aims to shed light on the potential association of music listening for optimizing creative cognition. This project is unique as, to the best of our knowledge, it is the first to experimentally test whether listening to specific types of music (classical music excerpts varying on mood and arousal), as compared to a silence control condition, facilitates creative cognition, as measured by divergent and convergent creativity tasks. Music listening can be easily integrated into daily life and may provide an innovative means to facilitate creative cognition in an inexpensive and efficient way in various scientific, educational and organizational settings when creative thinking is needed.

In the remaining part of the introduction we will put the study into context by providing theoretical background related to creativity, the effects of music listening on cognition, and on mood and creativity.

### Creativity

Creativity is usually defined as the generation of ideas, insights, or problem solutions that are original (i.e., new) and meant to be useful [16–18]. Creativity entails both divergent thinking and convergent thinking [19–21]. Divergent thinking involves producing multiple answers from available information by making unexpected combinations, recognizing links among remote associates, or transforming information into unexpected forms. A typical example of divergent thinking is idea generation, as measured in the Alternative Uses Task [19]. Although important, divergent thinking is only one component of creative cognition. Many scholars emphasize the need for an additional ability, convergent thinking; that is, the cognitive process of deriving the single best, or most correct, answer to a problem or question [22–24]. Convergent thinking emphasizes accuracy and logic, and applies conventional search, recognition, and decision-making strategies. As such, convergent thinking can easily be considered to be ‘uncreative’, but it may actually still require creativity as it is, for example, required in tasks where seemingly unrelated concepts have to be related, as measured in the Remote Associates Task [25]. In the current study, we investigate the role of music listening on the entire range of creative cognition, that is, on divergent and convergent thinking.

### Effects of music listening on cognition

Scientific interest in the potential benefits of music on cognition was sparked by the publication of an article that reported superior spatial abilities for participants who listened to music composed by Mozart compared to those who sat in silence [26]. It is assumed that these finding, known as the ‘Mozart effect’, can be explained by the perceiver’s arousal level and mood [27], which can affect performance on a variety of cognitive tasks [28]. Although extensive research has examined the impact of music on cognition, to the best of our knowledge there are only a very limited number of studies that have directly addressed the effects of music on *creative* cognition. Adaman and Blaney [2] published a study that used music to induce either a ‘depressed’, ‘elated’ or ‘neutral’ mood, followed by the subject completing a divergent creativity task (generating alternative uses for a household item). They found a significant effect for an increase in fluency (i.e., the number of uses generated) for both ‘depressed’ and ‘elated’ when compared with ‘neutral’ mood. For the originality of the generated uses, there was significant elevation in score for the ‘depressed’ mood, but not ‘elated’, while for flexibility no effect was found. Notably, however, the stimulus used was a set of 20 minute musical tapes which had been created by Pignatiello, Camp and Rasar [29], which were validated by observing heart rate and diastolic condition, and by measuring inter-rater reliability as to mood induction. Precisely what type of music was presented is not clear in this paper, and we will return to this issue later in the paper. Importantly, the study did not include a silence control condition, and measured only one aspect of creativity, divergent thinking. Ilie and Thompson [30] investigated participants’ performance on a creative insight task after exposure to music that varied in pitch height (high or low), rate (fast or slow), and intensity (loud or soft). Participants who listened to high-pitch music were more successful at solving the insight task than participants who listened to low-pitch music, and mediation analysis revealed that the effect of pitch height on insight task performance was fully mediated by the emotional valence participants associated with the music. Finally, Yamada and Nagai [31] conducted an experiment that compared participants who performed a creativity task while listening to happy music against a group who listened to a reading of the Japanese constitution. Participants were asked to generate new names for rice. Five examples of non-existent names, all ending with the word “hikari”, were provided. Names generated that ended with hikari were treated as conventional or typical ideas, reflecting fixed and constrained thought processes (i.e., convergent thinking), whereas names not ending with hikari were treated as unconventional or atypical ideas, reflecting free and unconstrained thought processes (i.e., divergent thinking). The quantity of convergent ideas was not affected by the sound being played to the participants prior to the creativity task, whereas the quantity of divergent ideas was larger for the group that listened to the happy music prior to the creativity task. The authors interpreted this to mean that being in a positive mood facilitates flexible thinking and consequently leads to the production of unconventional or atypical ideas.

### Mood and creative thinking

Extensive research has explored how mood states affect creative thought. In general, a positive mood appears to be associated to improvements in divergent thinking, but not convergent thinking (although this is not as clear) [32–33]. De Rooij and Jones [34] argued that the appraisal tendency perspective on moods (as opposed to the simple positive/negative perspective) gives a better explanation of the effect of mood on creativity. Similarly, research from De Dreu, Baas, and Nijstad [35] suggests that the arousal level (activating vs. deactivating) should be considered in the mood creativity link, as activating moods influence creative fluency and originality because of enhanced cognitive flexibility when mood is positive and because of enhanced persistence when mood is negative.

### Aims

In the current study, we aim to extend the sparse literature on the potential association of music listening for optimizing creative cognition. This project is unique, as it is the first to experimentally test whether listening to specific types of music, as compared to a silence control condition, facilitates creative cognition. Additionally, we structurally manipulate valence and arousal of the music, and we expose participants to the music excerpt while performing the creativity task and not before performing the creativity task. Importantly, creativity is measured by both divergent and convergent creativity tasks. Based on previous research, we hypothesize that positive and activating (i.e., happy) music facilitates divergent thinking. Due to scarce and inconsistent research findings, no specific hypotheses were formulated for the effect of calm, sad and anxious music on divergent thinking, as well as for the effect of music on convergent thought.

## Method

### Participants

A total of 155 participants (121 female; M_age_ = 22.30, SD_age_ = 5.24) gave written informed consent to participate in the study. The study was conducted according to the principles expressed in the Declarations of Helsinki and according to the principles of the institutional review board (Ethics Committee Faculty of Social Sciences, Radboud University, the Netherlands; ethical approval was not required as the research was not of a medical nature and there were no potential risks to the participants). Participants were recruited via the online research participation system (Sona) of Radboud University Nijmegen and gained either course credit of 0.5 hours or 5 € (in Iris cheques) for their participation.

### Design

A 2 x 5 mixed measures design was utilised with creative performance (divergent thinking, convergent thinking) as the within subjects variable and music condition (happy, sad, calm, anxious, silence) as the between-subjects variable.

### Materials

#### Music stimuli

Four pieces of music were selected, which were of different emotional valence (positive, negative) and arousal (low, high). The details of the music piece’s title, composer, and average root mean square (RMS) amplitude can be found in Table 1. In the control condition, participants completed the tasks in silence. The selected music pieces have been validated by earlier research to promote a particular mood [36]. Based on these validations, we refer to the five conditions applied in the current study as *calm* (positive valance, low arousal), *happy* (positive valance, high arousal), *sad* (negative valance, low arousal), *anxious* (negative valance, high arousal), and *silence* (no music induction).

**Table 1:** Details of the music stimuli played during the *calm*, *happy*, *sad*, *anxious* conditions.

Valence	Arousal	Condition	Title	Composer	Average RMS Amplitude	
Positive	Low	Calm	Carnival of the Animals: XIII. The Swan	Saint-Saens, Camille	-30.15 dB	
	High	Happy	The 4 Seasons, Op. 8, No. 1, RV 269, Spring–Mvt 1. Allegro	Vivaldi, Antonio	-27.05 dB	
Negative	Low	Sad	Adagio for Strings, Op. 11	Barber, Samuel	-37.23 dB	
	High	Anxious	The Planets: Mars, Bringer of War	Holst, Gustav	-27.96 dB

#### Creativity measures–Divergent creativity

Divergent thinking tests are open-ended tests. They are applied in approximately 40% of all creativity studies [37], and can be considered the most widely used creativity test [38–39]. One of the most frequently used and well-validated divergent thinking tests is the Alternative Uses Task (AUT) [19]. In the AUT participants are asked to list as many different and creative uses for a common object (in the current study a ‘brick’) as possible. Participants were instructed to type their ideas into a space provided on a computer display. They could type in an idea, and by hitting the Enter key, they could submit this idea and immediately receive a new opportunity to type in another idea. Participants were advised that their responses could be given in Dutch, English, or German. Creative performance during the AUT is reflected in an Overall divergent thinking (ODT) score—calculated by summing up participants performance on five indices of divergent thought: Fluency, Creativity, Originality, Usefulness and the Cognitive Flexibility of the ideas listed. These indices are discussed in the subsection below.

Fluency is a measure of creative production and represents the total number of ideas generated. To assign a fluency score, the number of ideas a participant listed are counted. Only complete (i.e., no unfinished) ideas were included in the fluency score.

Creativity is defined as the generation of ideas that are original (i.e., new) and meant to be useful [16–18]. A trained rater assigned all ideas a score on creativity, ranging from *not at all creative (= 1)* to *very much creative (= 5)*. A second trained rater assigned 30% of the listed ideas a creativity score. The inter-rater reliability of the ratings was calculated using a 2-way random intraclass correlation coefficient (ICC) analysis for consistency, and it was considered good (ICC_Creativity_ = .849). Per participant, a creativity sum score was calculated by adding the scores of the ideas a participant generated. Using a sum score is based on the quantity breeds quality assumption, that is, the idea that the more ideas are generated, the more ‘high quality ideas’ will be found among them [40]. In line with this reasoning, research has confirmed that creativity often increases with the number of ideas generated [41–43]. Moreover, by measuring creativity on the AUT by a sum score instead of by a mean score, we stay closer to the appraisal of creativity in real life. For example, audiences and stakeholders are more interested in the number of highly creative ideas or products somebody generated, than in the mean creativity of an individual’s work.

Originality is one of the core characteristics of a creative idea and refers to its uncommonness or infrequency [16–18, 44]. Common ways to score originality are applying the uniqueness scoring (i.e., counting the number of infrequent responses—mentioned by e.g. < 5–10% of the participant pool) or using judges to evaluate the responses for originality. While the uniqueness scoring allows for an objective scoring, a number of serious objections have been raised, for example, the problem that statistical infrequency does not account for the size of the participant pool and for the appropriateness of responses [45]. Using judges to evaluate all responses for originality [46, 47] is more subjective and time consuming, but it is considered a valid method due to the good inter-rater reliabilities that have been found [48]. In the current study trained judges were used to rate the responses for originality. One trained rater assigned all ideas a score on originality, ranging from *not at all original (= 1)* to *very much original (= 5)*. The second trained rater assigned 30% of the listed ideas an originality score. The inter-rater reliability of the ratings was calculated using a 2-way random intraclass correlation coefficient (ICC) analysis for consistency, and it was considered good (ICC_Originality_ = .781). Per participant, an originality sum score was calculated by adding the scores of the ideas a participant generated.

Usefulness is one of the core characteristics of a creative idea and refers to its effectiveness and practicality [49]. The trained rater assigned all ideas a score on usefulness, ranging from *not at all useful (= 1)* to *very much useful (= 5)*. The second trained rater assigned 30% of the listed ideas a usefulness score. The inter-rater reliability of the ratings was calculated using a 2-way random intraclass correlation coefficient (ICC) analysis for consistency, and it was considered good (ICC_Usefulness_ = .883). Per participant, a usefulness sum score was calculated by adding the scores of the ideas a participant generated.

Cognitive Flexibility manifests itself in the use of different cognitive categories and perspectives [16, 25], and it can be measured by the number of distinct idea categories a participant uses. For example, when asked to list alternative uses for a brick, the answer “build a house, build a wall, build a garage” would lead to a cognitive flexibility score of one, as all ideas are assigned to the category ‘building something’, whereas “build a house, break a window, use it as a pen holder” would lead to a score of three, as the ideas are assigned to three different idea categories (‘building something’, ‘destroying something’, ‘utensil’). Two trained raters worked together on developing the list of idea categories for the brick. Thereafter, one of the trained raters assigned each idea to a category from the predefined list of idea categories. Raters were blind to conditions, both when (i) developing the list of categories and (ii) when assigning the ideas to the pre-defined categories. Finally, per participant the total number of distinct idea categories used was calculated.

The Overall divergent thinking score (ODT) was calculated by adding up the five divergent thinking indices described above.

#### Creativity measures–Convergent thinking

Convergent thinking tests measure whether a participant succeeds in coming up with the best, well-established, or correct answer to a problem where an answer readily exists [50]. In the current study convergent thinking is tested by means of the Idea Selection Task, Remote Associates Task and Creative Insight Task.

During the Idea Selection Task, participants were presented with 10 inventions for the kitchen, and were asked to select the three most creative inventions from this list of pictures. The instructions during the task advised participants that a creative invention should be both original and useful. Once participants had selected the three inventions they considered most creative, these items were re-presented and participants had to arrange the three items in order of creativity. Each selected invention (1st, 2rd and 3rd most creative) was assigned a creativity score, based on expert ratings, resulting in an Idea Selection Score (the creativity score of the idea selected as most creative idea) and a Mean Idea Selection Score (the mean creativity score of the ideas selected as most, second most and third most creative idea).

During the Remote Associates Task (RAT, adapted from [51]), participants were presented with a list of ten three-word combinations. Their task was to generate a fourth word which connected the three seemingly unrelated words (Example 1: bar–dress–glass; fourth word: cocktail; cocktail bar, cocktail dress, cocktail glass). Participants could choose prior to the commencement of the task whether they would complete the task in English or in Dutch, and this determined whether the English or Dutch version of the target words were displayed.

During the Creative Insight Task, participants were presented with two insight problems (i.e., the ‘Two-string problem’ and the ‘Duncker candle problem’). To solve these problems, one has to use a displayed object in an unfamiliar manner. For example, in the two-string problem, one has to tie together two strings hanging from the ceiling. However, the strings are arranged so far apart that they cannot be reached at the same time. The solution requires setting the string in motion as a pendulum. The problems were displayed on screen, accompanied by an image, and participants had to type their solutions to each problem in the space provided.

#### Questionnaires

To obtain data about pre-existing mood, participants were presented with 22 emotion-words that varied on mood and arousal (positive-activating, positive-deactivating, negative-activating, negative-deactivating; for example, happy, calm, angry, sad; see [33]) and had to indicate how much of each emotion they had experienced since they got up on the day of participating using a 5-point Likert scale ranging from 1 (*not at all*) to 5 (*very much*). Each of the 22 mood items were presented in English and accompanied with the term in Dutch.

To assess preference for the musical excerpts, participants were asked to indicate how much they liked the music using a 7-point Likert scale ranging from 1 (*not at all*) to 7 (*very much*), and whether they were familiar with the music (*list title and composer*). In addition, they were asked to describe the emotions evoked by the music, on 7-point Likert scales they had to indicate the valence of the music (*very negative* to *very positive*) and the arousal level of the music (*very deactivating* to *very activating*). Prior experience with being creative (“Do you have experience with being creative?” c*hoice options*: *no experience*, *some experience*), and with music (“Do you have experience with making music / the music industry?” c*hoice options*: *no experience*, *some experience*) was also ascertained. Several demographic items established the gender, age, and nationality of participants, as well what participants were studying at the time of their participation.

### General procedure

Consenting participants were randomly assigned to one of the experimental conditions (*calm*, *happy*, *sad*, *anxious*; for more information, see Table 1) or a *silence* (control) condition. First, participants were informed that they will perform four different tasks and several questionnaires. Between each task, there was a 20 seconds break, and a countdown timer was displayed to prepare participants for the following section. Participants in the music conditions were, moreover, told that they are exposed to music right before and during the completion of each of these tasks. In the music conditions, exposure to the music occurred for 15 seconds prior to the task appearing, in order for participants to “get into” the music. During these 15 s the screen was blank. Once the music had played for 15 seconds, the task replaced the blank screen and the music continued playing. The music was played through Sony MDR7505 headphones at a Windows-PC. The volume was set at a comfortable level, numerically standardised across each computer, and then maintained for the duration of the experiment.

First, participants completed the mood questionnaire. Thereafter, creative task performance was measured using the Alternative Uses Task, the Idea Selection Task, the Remote Associates Task, and Creative Insight Tasks, in that order. Each task was set to a total of 3 minutes, such that participants could spend up to three minutes on each task. If they completed the idea selection, RAT, or Creative Insight tasks in less than three minutes, they could proceed to the next section. After completing the four creativity tasks, participants completed the music and demographics questionnaires. At their completion of the study, participants were debriefed and awarded course credit or payment for their participation.

Participants completed the study individually in cubicles, and the lights were slightly dimmed so that the level of brightness was equal across the cubicles used. During the completion of the study, the experimenter was sitting in a room outside of the cubicle, and participants could ask the experimenter for help if any instructions were unclear.

## Results

### Questionnaires

#### Mood and arousal

There was no difference between conditions on participants’ mood and arousal level prior to the experiment (positive-activating: *F*(4, 150) = 1.68, *p* = .158, η_p_^2^ = .043, positive-deactivating: *F*(4, 150) = 0.81, *p* = .518, η_p_^2^ = .021, negative-activating: *F*(4, 150) = 1.41, *p* = .233, η_p_^2^ = .036, negative deactivating: *F*(4, 150) = 1.30, *p* = .273, η_p_^2^ = .034).

#### Music ratings

There was no difference between the four music conditions on liking of the musical excerpts (*F*(3, 124) = 1.280, *p* = .285, η_p_^2^ = .031), and on familiarity with the musical excerpts (χ(3, *N* = 124) = 5.63, *p* = .131). As expected, a main effect was found between music conditions on the experienced valence of the music (*F*(3, 120) = 10.59, *p* < .01, η_p_^2^ = .209) and on the experienced arousal of the music (*F*(3, 120) = 25.51, *p* < .001, η_p_^2^ = .389). Participants in the happy music condition (*M =* 5.65, *SD* = 1.11) assigned the music a significantly higher *valence* score than did participants in the calm (*M =* 4.52, *SD* = 1.50), anxious (*M =* 4.13, *SD* = 1.61) and sad (*M =* 4.59, *SD* = 1.52) music condition (all p’s < .001). Participants in the happy music condition (*M =* 5.23, *SD* = 1.43) assigned the music a significant higher *arousal* score than did participants in the calm (*M =* 3.35, *SD* = 1.14) and sad (*M =* 4.40, *SD* = 1.69) music condition (all p’s < .001). Moreover, participants in the anxious music condition (*M =* 4.13, *SD* = 1.61) assigned the music a significantly lower arousal score than participants in the calm condition and the sad music condition (all p’s < .001).

#### Experience with music and creativity

The five conditions did neither differ on musical experience, χ(3, *N* = 155) = 7.03, *p* = .534, nor on experience with creativity, χ(3, *N* = 155) = 11.67, *p* = .166.

#### Creativity

In this study, creativity has been assessed in terms of divergent and convergent thinking abilities.

#### Divergent thinking

Divergent thinking is usually associated with the ability to generate new ideas and new perspectives. In this study, it was tested using the Alternative Uses Task (AUT) [19]. Six of the 155 participants were excluded from the analyses, as they did not understand the AUT (task instruction: “List as many different and creative uses for a brick; 2 participants listed feelings related to the music they were exposed to, 1 participant listed associations with a brick, 1 participant mentioned sayings about a brick, and 2 participants listed brick unrelated ideas, e.g., ‘technological development’, ‘speed’, ‘peace’).

The AUT variables Overall divergent thinking (ODT), Fluency, CreativitySum, OriginalitySum, UsefulnessSum, and Flexibility were skewed (|2 × SE SKEW| < Skewness statistics). The skweness was corrected by applying a square root transformation. The transformed and untransformed values correlate highly (> .95), and the transformation of values did not change the general interpretation of the findings. ANOVA’s were conducted on the transformed data, and the t-test was conducted on the non-transformed data. Descriptive statistics of the non-transformed data are mentioned.

The correlations between the overall divergent thinking score (ODT) and the five indices of divergent thinking are presented in Table 2.

**Table 2:** Correlations between the five divergent thinking indices and overall divergent thinking.

Measures	1	2	3	4	5	6
**1. SumCreativity**	—	0.977*	0.857*	0.873*	0.924*	0.967*
**2. SumOriginality**		—	0.847*	0.872*	0.921*	0.963*
**3. SumUsefulness**			—	0.832*	0.973*	0.954*
**4. Flexibility**				—	0.874*	0.904*
**5. Fluency**					—	0.985*
**6. ODT**						—

Remark: Pearson correlations between transformed variables (SQRTtrans).

*p < .001.

ODT = Total divergent thinking.

**Fig 1:**
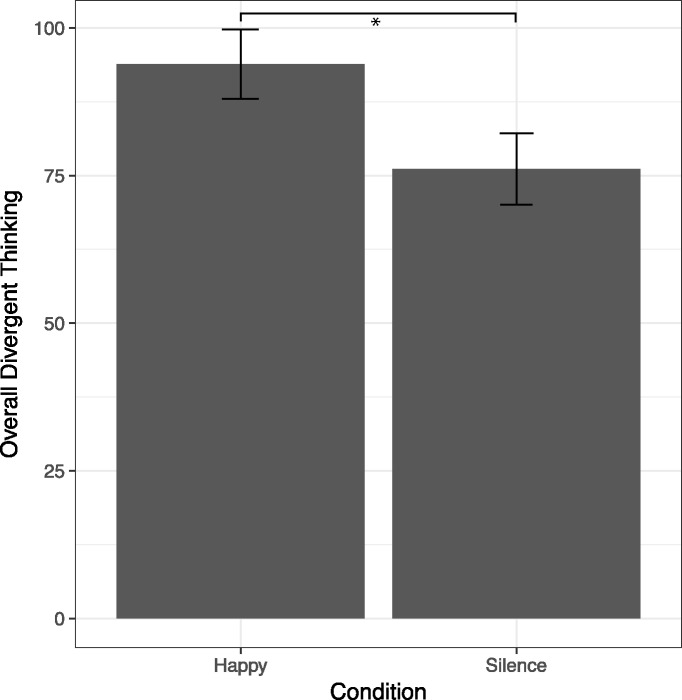
Overall divergent thinking (ODT) score. Mean of the Overall divergent thinking (ODT) score during the Alternative Uses Task in the happy music and silence control condition. Error bars represent Standard Error of the Mean. Significant differences between conditions at p < .05 are represented with *.

Our main hypothesis was that listening to happy music, as compared to a silence control condition, facilitates divergent thinking. An independent-samples t-test was conducted to compare the happy music condition with the silence control condition on overall divergent thinking (ODT). There was a significant difference on ODT between the happy music (M = 93.87, SD = 32.02) and silence (M = 76.10, SD = 32.62) conditions, t (57) = 2.110, p = .039, d = .550. The results suggest that listening to happy music increases performance on overall divergent thinking (Fig 1).

For the comparison between the five conditions on overall divergent thinking (ODT) and the five indices of divergent thinking no specific predictions were formulated. For exploratory reasons, for each of the six dependent measures a one-way ANOVA was conducted. A difference between conditions was observed for ODT, Fluency, CreativitySum and OriginalitySum, but with Bonferroni correction (alpha level at .05/6 = < .0083) these differences were not significant (p = .039, p = .036, p = .023, p = .017; respectively) and, therefore, no post hoc comparisons were conducted.

### Convergent thinking

Convergent thinking is assessed by a Selection Task, a Remote Associates Task, and Creative Insight Tasks.

#### Idea selection

One of the 155 participants was excluded from the analyses due to computer problems during the selection task. An ANOVA with the Idea Selection score as dependent variable and condition as independent variable revealed no significant difference between conditions (*F(4*, *149) = 0*.*718*, *p =* .*581*, η_p_^2^ = .019). In addition, no difference was found between conditions for the mean Idea Selection Score of the three selected ideas (*F*(4, 149) = 0.81, *p* = .521, η_p_^2^ = .004).

#### Remote associates

A frequency analysis showed that 12 participants (7,7%) did not solve any of the 10 RAT word-pairs, 92,3% of the participants had solved between 1 and 9 RAT word-pairs, and none of the 155 participants (0%) solved all 10 RAT word-pairs. Thus, there was sufficient variation in task performance, and an ANOVA with the number of RAT items solved as dependent variable and condition as independent variable was conducted. The analysis revealed no significant difference on RAT performance between conditions (*F*(4, 150) = 0.36, *p* = .839, η_p_^2^ = .009).

#### Creative insight

A frequency analysis showed that only 2 (1.3%) of the 155 participants solved both creative insight problems, 32 participants (20,6%) solved one of the creative insight problems, and 121 participants (78,1%) solved none of the two creative insight problems. Due to the low variation in task performance, this task was excluded from further analysis.

## Discussion

This study has investigated creativity in the context of music listening. In an experimental design, we tested whether listening to specific types of music (four types of music that varied on valence and arousal), as compared to a silence control condition, is beneficial for creativity. The main conclusion of the results we obtained is that listening to ‘happy music’ (i.e., classical music that elicits positive mood and is high on arousal), as compared to a silence control condition, is associated with an increase in divergent thinking, but not convergent creativity.

Why does listening to ‘happy music’ enhance divergent creativity? The dual pathway to creativity model [52] argues that creative ideation is a function of persistence and flexibility, and that situational variables can influence creativity either through their effects on persistence, on flexibility, or on both. For example, one can rely on effortful in-depth exploration of a few possibilities and perspectives. However, when getting stuck in a rut, it can be helpful to, instead of digging deeper, dig elsewhere. Hereby, as illustrated in the following problem, a flexible thinking style can be beneficial: In developing countries neonatal death rates are high due to unavailable resources to repair high-tech incubators. Solutions found by ‘digging deeper’ could be to further develop the technology so that incubators do not break down, or to train locals to repair these high-tech devices. A creative solution generated by thinking flexibly and by ‘digging elsewhere’ is to design an incubator from car parts, as developing countries have mechanics who possess knowledge about car equipment. A flexible thinking style is not limited to one particular creative field [25], but is equally valid for artistic, verbal and scientific creativity, and may have helped participants in the current study to come up with more creative ideas on the divergent thinking task used in the current study. Interestingly, as opposed to divergent thinking, listening to happy music did not lead to higher performance on the convergent creativity tasks—despites earlier findings showing that convergent thinking can be facilitated [53]. The increase in divergent but not convergent thinking after listening to happy music may be explained by the fact that the convergent tasks rely less on fluency and flexibility, but on finding one correct answer.

The current study provides support to the creative cognition model of creativity (for example [9]), which states that individual differences in creativity can be explained by variations in the efficiency of cognitive processes underlying creativity, and to the idea that creative thinking can be enhanced [10, 12].

### Limitations of the current study and suggestions for future research

As mentioned earlier, we assume that the performance difference on the divergent and convergent creativity tasks in the happy music condition can be ascribed to difference in task demands of divergent (generating as many ideas as possible) and convergent (finding one correct answer) creativity. However, given that when performing the convergent creativity tasks, participants were exposed to the happy music for the second time, we cannot rule out habituation as an alternative explanation for the performance difference. In the current study, we compared the effect of four different types of music (high vs. low arousal, and positive vs. negative mood) on creative performance in a between-subject design, that is, participants were either assigned to one of the four music conditions or to the silence control condition. Given that we already employed 5 conditions, divergent and convergent creativity was used as a within-subject task, that is, each participant first performed the divergent creativity task while listening to the specific music excerpt (or, in the control condition, performed the task in silence) and, thereafter, performed the convergent creativity tasks while listening to the same music excerpt (or, in the control condition, performed the task in silence). As the convergent tasks can be frustrating if the tasks are not solved, and hereby influence subsequent task performance, the convergent tasks were always performed after the divergent task. Future research could employ only one type of music, happy music, and could include creativity task (divergent vs. convergent) as a between-subject factor. If, as we assume, task demands and not habituation account for the difference in findings between the divergent and convergent tasks, a beneficial effect of music listening should be observed for the divergent but not for the convergent creativity task.

As many people benefit from creative thinking, and we wish these results to have an as wide applicability as possible, our participants were primarily college age adults and not people already involved in creative work. However, the participant sample had a high education level and a relatively high proportion of females and westerners, which could limit the ecological validity of this study. Findings of a meta-analysis [12] suggest that creativity enhancement may be more effective in organizational than academic settings and may have greater effects on men than on women. Considering that this study relied on a population and setting for which the a priori chance of finding a stimulation effect was not high, the ecological validity and generalizability of the current findings may be enhanced. Nevertheless, it is still unknown what impact such training would have on eastern participants and on other age groups, for example, school-aged children and elderly people. Future research could examine the effect of music listening in eastern cultures and for other age groups. Moreover, follow-up research could study the moderating role of educational background, such as music experience. Given that even for those not genuinely interested in music an effect of music listening was found on creative thinking, we expect this effect to be even stronger for musicians.

While the current study provides evidence that listening to ‘happy music’ enhances creativity on a divergent thinking task, some additional questions related to the music played could be addressed in follow-up research. First, the music stimuli used in this experimental procedure were chosen for their effectiveness in producing the four emotions nominated [36]. In future research, rather than being inferred from the music condition presented, mood and arousal could be measured using extensive self-report or other relevant measurement methods (e.g., observing heart rate and diastolic condition). This would be likely to provide higher accuracy and may show stronger results between music type and the creativity results. In addition, future research could investigate two parameters that may be relevant for the link between music listening and enhanced creative performance, that is, liking of the music and familiarity with the music. Whereas in the current study there was no difference between the four music conditions on liking of the music and on familiarity with the music, future research could manipulate these effects by asking people to select their most favourite music excerpt, or by selecting a music excerpt they are not familiar with. Second, future research could explore the effect of different music genres (e.g., the effect of non-classical music that is high on positive mood and arousal, such as dance or trance), or one could extend the study by testing the effects of different sounds (e.g., ambient wind chimes, grinding machinery) on creative performance. Third, future research could explore whether it is necessary to listen to music while performing the creativity task, or whether music listening prior to the creativity task is sufficient. With regard to the creativity measures, in the current study creative performance was measured by well-validated and frequently used divergent and convergent thinking tests. In follow-up studies it could also be interesting to assess creativity in terms of real world creative achievement, and to investigate whether music listening is particularly effective for specific creativity domains, for example, artistic creativity or scientific creativity. Importantly, the current study does not allow any conclusions to be made about the long-term effects of music listening on creativity. In future research, a follow-up measure could be included to gain information about the duration of the effect, or whether a maintained effect can be developed by frequent music listening.

### Conclusions

Creativity is one of the most important cognitive skills in our complex, fast-changing world. In the past decades, various techniques to enhance creative thinking have been developed and tested. However, many of the currently available creativity enhancement techniques have to be trained and explicitly communicated, which can be time and cost intensive. Employing music listening as a means to stimulate creativity has yet, remained relatively unexplored—despites earlier scientific studies demonstrating a beneficial effect of music on human cognition. The current project aimed to shed light on the potential association of music listening for optimizing divergent and convergent creativity, and demonstrated that listening to ‘happy music’ (i.e., classical music that elicits positive mood and is high on arousal) is associated with an increase in divergent thinking, but not convergent thinking. In addition to the scientific contribution, the current study may provide important practical implications—music listening may be useful to promote creative thinking in inexpensive and efficient ways in various scientific, educational and organizational settings when creative thinking is needed.
